# The Fate of the Missing Spores — Patterns of Realized Dispersal beyond the Closest Vicinity of a Sporulating Moss

**DOI:** 10.1371/journal.pone.0041987

**Published:** 2012-07-27

**Authors:** Niklas Lönnell, Kristoffer Hylander, Bengt Gunnar Jonsson, Sebastian Sundberg

**Affiliations:** 1 Plant Ecology, Department of Botany, Stockholm University, Stockholm, Sweden; 2 Department of Natural Sciences, Engineering and Mathematics, Mid Sweden University, Sundsvall, Sweden; 3 Department of Plant Ecology and Evolution, Evolutionary Biology Centre, Uppsala University, Uppsala, Sweden; 4 Swedish Species Information Centre, Swedish University of Agricultural Sciences, Uppsala, Sweden; Norwegian University of Science and Technology, Norway

## Abstract

It is well-known that many species with small diaspores can disperse far during extended temporal scales (many years). However, studies on short temporal scales usually only cover short distances (in, e.g., bryophytes up to 15 m). By using a novel experimental design, studying the realized dispersal, we extend this range by almost two orders of magnitude. We recorded establishment of the fast-growing moss *Discelium nudum* on introduced suitable substrates, placed around a translocated, sporulating mother colony. Around 2,000 pots with acidic clay were placed at different distances between 5 m and 600 m, in four directions, on a raised bog, with increased pot numbers with distance. The experiment was set up in April–May and the realized dispersal (number of colonized pots) was recorded in September. Close to the mother colony (up to 10 m), the mean colonization rates (ratio of colonized pots) exceeded 50%. At distances between 10 and 50 m colonization dropped sharply, but beyond 50 m the mean colonization rates stabilized and hardly changed (1–3%). The estimated density of spores causing establishments at the further distances (2–6 spores/m^2^) was realistic when compared to the estimated spore output from the central colonies. Our study supports calculations from earlier studies, limited to short distances, that a majority of the spores disperse beyond the nearest vicinity of a source. The even colonization pattern at further distances raises interesting questions about under what conditions spores are transported and deposited. However, it is clear that regular establishment is likely at the km-scale for this and many other species with similar spore output and dispersal mechanism.

## Introduction

Dispersal, especially long-distance dispersal, is of utmost importance for understanding patterns and processes like species distributions, metapopulation dynamics and range expansions. This includes responses to climate change. Dispersal is thus continuously rendering attention from many biologists [1]–[3]. However, empirical data on dispersal distances, especially at longer distances and what proportion of diaspores that really establish at those distances, are still badly needed in order to evaluate model predictions and formulate new hypotheses [4], [5]. The problem has been approached both by direct and indirect methods. However, due to methodological constraints the number of direct studies is low as they become increasingly laborious when distances increase.

Organism with small diaspores, like fungi, lichens, ferns, bryophytes, diatoms, bacteria, yeasts, as well as microscopic animals, are often more widely distributed than organisms with larger diaspores. However, the total ubiquity of microorganisms, as simplified by the 19th century expression “Everything is everywhere but the environment decides”, may be highly exaggerated [6]. Over large temporal scales, dispersal inferred from distributional patterns seems to be efficient for many species of bryophytes, lichens and ferns also at very large spatial scales [7]. However, there are biogeographical studies that both support and contradict the intercontinental dispersal of organism with small diaspores [6].

Bryophytes dispersing with spores is an example of a species group where there is no unequivocal and general answers regarding the dispersal capacities and distances. Over moderate temporal scales (50–200 years) many bryophytes manage to disperse effectively many kilometres, as shown by studies of bryophyte colonization on introduced substrata and newly created habitats [8]–[10], intraspecific genetic structure [11] and patterns of species composition on land-uplift islands [12]. Hylander [13] found no positive effect of proximity to sources on the colonization rates up to 80 m from diaspore sources in forest bryophytes after 40–60 years, which may indicate that the realized dispersal at this temporal scale is efficient. Nevertheless, other indirect studies inferring dispersal distances from distribution patterns of genes or species on a non-specified temporal scale show that at least some species are dispersal limited. For example, the occurrence and abundance of epiphytic bryophytes and lichens seem to be correlated to connectivity to occupied trees and stands within landscapes of a few kilometres of extension [14]–[16]. However, even if a species regularly disperse to a site, the establishment rate can depend on the amount of diaspores reaching that site, as indicated by results from studies of polyporous fungi [17], [18]. Norros et al. [19] suggested that many forest fungi can be dispersal limited due to a very low establishment probability.

The results from direct studies of bryophyte diaspore dispersal do, at least superficially, contrast to most of the studies that use indirect measures, referred to above. Several direct studies have been performed but the distances covered have been restricted to 2–10 m [20]–[24]. All studies show that the number of spores trapped decrease rapidly with distance, to low levels at the outer perimeter of the experiments. However, they also demonstrate that a considerable proportion of the spores (30–97%) cannot be accounted for within the studied distances and hence have dispersed further away.

One challenge is thus to quantify how dispersal behaves at further distances from a point or patch source. Different statistical (phenomenological) models, such as the negative exponential, inverse power law, Weibull, Gaussian, Laplace, Wald, as well as diffusion models have been fitted to the density versus distance relationship in various dispersal experiments [3], [19], [25]. One important difference between these functions is the thickness of the tail (which has implications for the inference of dispersal capacity at longer distances), where for example the inverse power law predicts a slower decay rate than the negative exponential. Over the short distances evaluated in the datasets on bryophyte dispersal (mentioned above), the inverse power law generally fitted better than the negative exponential to the density versus distance patterns [23]. The shape of a dispersal curve could thus also indicate something about the dispersal mechanisms. Among organisms with small diaspores, that can be caught by turbulent winds, it is likely that the negative exponential function underestimates the dispersal distances which are thus better described by a power law function [26].

Inherent traits such as size, shape and weight of the diaspores determine the settling speed and the time in the air [27] as well as the release height of the diaspores [28], which strongly influences the probability that the diaspores are caught by the wind and are dispersed further distances [29]. Different authors have suggested that diaspores of sizes below certain thresholds are significantly more easily dispersed than above those thresholds. Suggested threshold values are among others: From 1–2 mm, cf. [30], to 20 µm [31], [32].Not horizontal wind speeds *per se* but rather vertical air movements caused by frictional turbulence [33] and thermal upheaval [34], are prerequisite mechanisms for dispersing diaspores over long distances. If the initial gravity force is overcome, the exact distance to the source might not be so important and meteorological factors will become increasingly important to predict dispersal distances.

The objective of this study was to increase the understanding of dispersal patterns across landscapes of organisms with small (around 20 µm) wind-dispersed diaspores. We asked if spores regularly disperse and establish over many hundreds of meters and what the spatial distribution of establishments can tell us about the dispersal mechanisms involved. We used a novel study system of a rare and substrate-specialized, short-lived acrocarpous moss, where c 2000 pots with its target substrate were placed at different distances from a patch source with a sporulating moss. It allowed us to extend the measurements of dispersal distances by almost two orders of magnitude compared with previous similar work on bryophytes. We then studied the realized dispersal including both the transport of diaspore and the establishment of the gametophyte, allowing us to detect also low densities of spores and exploring much longer distances than in previous studies. The spatial and temporal scales in this study are relevant for many fugitive and colonist species, sensu [31], dependent on short-lived substrates.

## Materials and Methods

### The study organism

*Discelium nudum* (Dicks.) Brid. is a rare acrocarpous moss which inhabits newly exposed, acidic clay or silt along water courses and ditches, primarily in agricultural landscapes. It is sometimes found also along forest roads or in wheel tracks in clear-cuts. The spores are spheroid with an average diameter of 24.6 (21.8–30.1) µm [35] and are released during late April—mid May in Sweden [36]. After a spore has germinated, an extensive protonema develops and after a few weeks several shoots are formed from the protonema. The up to 2 mm tall shoot consists of only a few leaves surrounding the antheridia or archegonia but are easily distinguished from other species at this stage. Fertilization occurs during the summer [36]. The sporophyte develops from the fertilized archegonium during autumn and releases mature spores the next spring. The sporophyte consists of a horizontal capsule (0.7–1.0 mm×0.5–0.8 mm) on a seta of up to 3 cm (personal observation) and the opening is surrounded by a double peristome [37]. The species is classified as pseudo-dioecious, i.e. the female and male shoots develop from the same protonema [38], [39]. Hence one spore can result in many shoots and from every female shoot may one capsule origin and contribute to the dispersal. It is not possible to determine if colonization is caused by one or several spores as protonemata from different spores may intermingle and result in a continuous protonema mat.

Nomenclature of mosses follows Hill et al. [40].

### Study design and data collection

**Figure 1:**
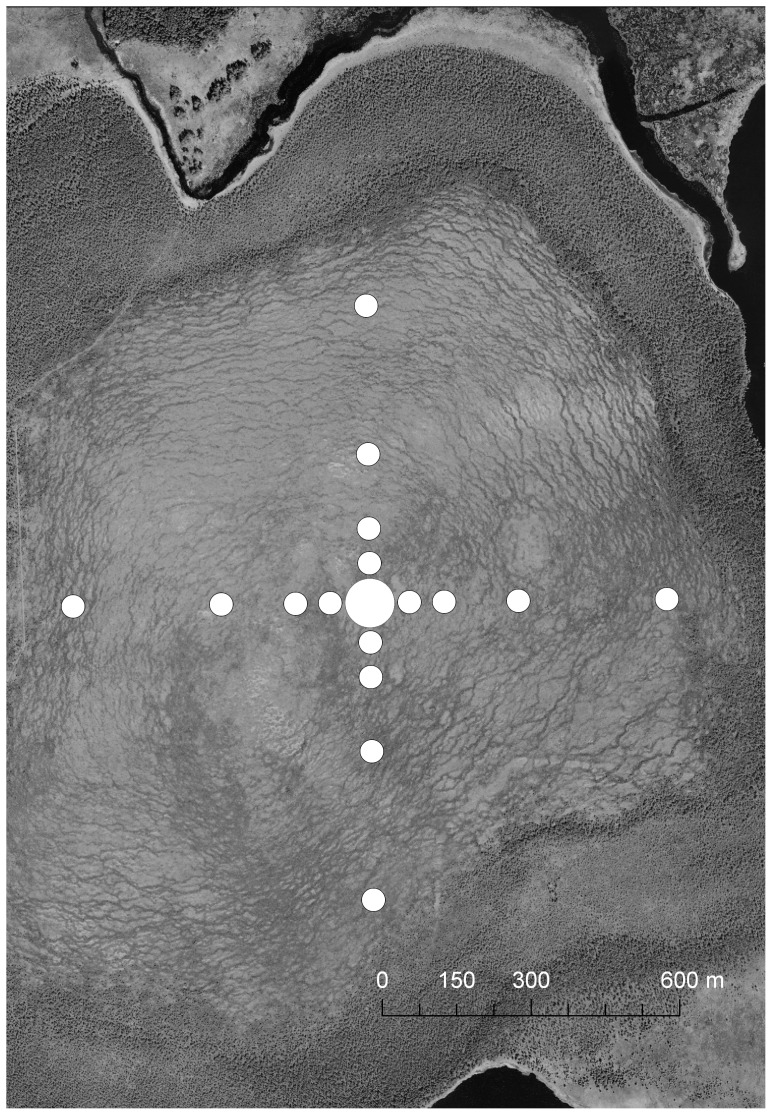
Map of the field site and study design at Jordbärsmuren. The mother colony was situated in the centre: pots were placed at 1, 5, 10, 30, 50, 80, 150, 300 and 600 m in each direction at in total 36 sampling stations. Note: The area with the sampling stations from 50 m and inwards are represented by a large white circle. Background aerial photographs: © Lantmäteriet Gävle 2011 (Permit I 2011/0094).

The experiment was performed in east central Sweden in 2010. The area was chosen because the model species is rare here. The field site, Jordbärsmuren, is a 2 km^2^ open raised bog in the southern part of the province of Gästrikland (N60.311°, E16.941°). The clays in the area might be suitable for *Discelium*, but there was no area with clay within 600 m of the outermost sampling stations (Fig. 1). The nearest colony of *Discelium* that could be found in the vicinity during the autumn after the experiment was a very small population (less than 100 young capsules) 10 km away and there are no historical records within 20 km from this site (collections from the following herbaria: Stockholm (S), Uppsala (UPS), private herbaria).

Clay (pH = 6.1) was dug up, with help of an excavator, from a depth of 1–2 m (5 April 2009) at a site *c* 35 km NW of Jordbärsmuren. Great care was taken to minimize the risk of contamination throughout the handling of the clay. After the surface soil was removed, the dipper of the excavator was cleaned with towels to avoid contamination from any kind of diaspores. The clay was transported to Stockholm and stored in plastic boxes within sealed plastic bags. The clay was distributed into plastic pots (7×7×7 cm) inside a greenhouse. The pots were stored and transported to the mire in plastic boxes covered by lids. The pots with clay were installed in the field on 19 and 20 April 2010. In total 2100 pots were positioned at 36 sampling stations at 1, 5, 10, 30, 50, 80, 150, 300 and 600 m in the four cardinal directions from the central point (Fig. 1). The number of pots was increased with distance from the centre in proportion to the total area at each distance; from 6 pots per station at 1 m to 252 per station at 600 m (Table 1, Table S1). As the number of pots was rather low at 1 and 5 m, these distances were pooled in the analysis. Approximately 10 000 ripe but not dehisced sporophytes (see below for the procedure for estimation of sporophyte numbers) were brought from a site near the city of Umeå 430 km north of the study area. At the central point, the mother colony of 11 dm^2^ was translocated into a small hummock approximately 1 dm above the lowest part of the surroundings (two thirds on 6 May and one third on 15 May). The colonizations of *Discelium* were counted on the 8 and 9 September. At some of the sampling stations, the number of colonizable pots was markedly reduced (up to 50% at some sampling stations) due to activities of animals that tipped/destroyed some of the pots (See Table 1, Table S1). Still, however, >1800 pots were available for evaluation.

**Table 1:** Mean colonization rate and spore density per distance.

Distance (m)	No of stations	No of pots	Colonizable pots	Colonized pots	Colonization rate (mean ± SD)	Spore density (mean ± SD; m^−2^)
1–5	8	48	48	40	0.83±0.20	339±123
10	4	32	31	20	0.64±0.08	214±48
30	4	52	43	6	0.16±0.19	40±46
50	4	84	75	1	0.01±0.03	3±6
80	4	136	114	3	0.03±0.02	6±4
150	4	252	193	2	0.01±0.01	2±3
300	4	504	422	7	0.02±0.02	4±4
600	4	1008	890	13	0.01±0.02	3±5

For each distance the number of sampling stations, the total number of pots put out, those who remained colonizable, colonized pots, mean colonization rates (ratio of colonized pots) and calculated density of spores, based on the number of colonised pots per distance from the mother colony.

To detect background deposition, 252 and 233 pots were established at two mires. The first mire is situated 7 km from Jordbärsmuren and was checked for colonizations in the field in September 2010. The second mire is situated 36 km from Jordbärsmuren, but 8 km from another translocated colony of *Discelium* (not used in this experiment) and was checked in the field in June 2011.

The pH of the clay used in the experiment was measured by suspending 50 g clay in 5 cl of distilled water. Then this suspension was mixed and left for 2 minutes before measurement with a pH meter (Metrohm 744).

The number of released spores from the mother colony was estimated as follows: the content of one capsule was emptied in 10 ml of water. Then a drop of the well-mixed spore suspension was applied to a haematocytometer (Bürkner counting chamber) and the number of spores was counted in a known volume of water within the chamber. Counting was repeated 10 times for each of 6 capsules from 3 sites. The area occupied by *Discelium* in the central colony was measured from a digital photograph and multiplied by an estimation of the capsule density to obtain the number of capsules in each translocated mother colony. Finally, the number of spores per capsule (mean ± SD) was multiplied by the estimated number of capsules.

All necessary permits were obtained for the described field studies. Permission was granted from the county administrative board of Gävleborgs län for performing the experiment on the site. The field studies did not involve endangered or protected species.

### Analyses

We used an indirect method to estimate the density of deposited spores which resulted in establishments at each sampling station. If one out of 100 pots were colonized in such a station it seems likely that this colonization was caused by only one spore. If, on the other hand, 99 out of the 100 pots were colonized it is unlikely that only one spore per pot had caused the colonization. Rather it is more likely that most of the pots were colonized by several spores since only one pot was not hit by any spore (assuming equal colonization probability of all spores). If we assume that the colonizations reflect the deposition, we can use the ratio of uncolonized vs. colonisable pots to obtain an estimation of number of deposited spores. Presuming that the spores were randomly deposited at each sampling station the frequency of the number of spores deposited on a specific area (in this case a pot) at one sampling station can be described by a Poisson distribution.

Because we knew the proportion of pots without colonizations we could derive the parameter λ (equation 1), which is the only unknown parameter in a Poisson distribution denoting the mean (and also the variance).


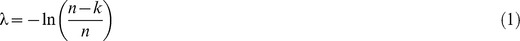


where:

λ = the mean number of spores per pot (out of the number of pots at one sampling station)
*n* = the number of pots at one sampling station
*k* = the number of pots with colonization at one sampling station

Equation 1 thus gives us the mean number of colonizations per pot under a specific outcome of number of colonized versus non-colonized pots at a sampling station. Based on the area of the pots, the spore density at the sampling station can be calculated.

However, if all or none of the pots are colonized at a given sampling station, it is actually impossible to calculate the mean of the Poisson distribution. When there were no colonizations the density was set to 0 and when all the pots were colonized the density was set to the same value as if all but one pot was colonized. This thus underestimates the density at those sampling stations where all pots were colonized, but provides the best possible estimate.

We fitted an inverse power function to the mean number of estimated deposited spores at each distance, assuming a similar deposition pattern in all directions, to explore the slope of the function. We also analysed if there were any indications of threshold patterns indicating a mixture of dispersal mechanisms acting together, cf. [41]. If an inverse power curve, describing the density of spores at different distances from a source (a probability density function), has an exponent larger than −1, the number of spores reaching a given distance interval increases with increasing distance taken into account that the area increases further away from the center. If such a pattern is found it could indicate that there is a mechanism transporting more diaspores further away, the presence of a background deposition or that the dispersal is higher at recorded than unrecorded distances.

To estimate the total number of spores that had dispersed and caused colonization up to 1 km we integrated the fitted inverse power law curve with the following equation, cf. [23]:


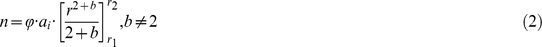


where:

*n* = spore number
*φ* = angle of the sector in radians (here 2π)
*a_i_* = spore density at 1 m from the centre of the source
*b* = the rate of decline with distance (in m) from the source
*r_1_* = the inner radius (here 1 m)
*r_2_* = the outer radius (here 1000 m)

For the regression, the software R version 2.14.1 [42] was used.

## Results

**Figure 2:**
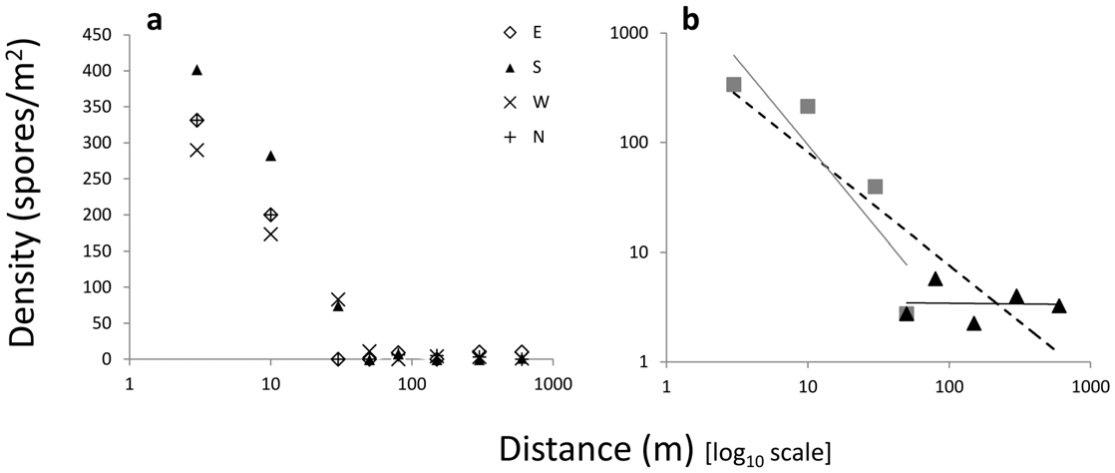
The estimated spore densities, based on the observed number of colonised pots, at different distances. a. Spore densities (on a linear scale) for the four cardinal directions (distances on a logarithmic scale). b. The mean spore densities at each distance (in log-log space). Power trend lines are shown both for the full data (hatched) and for distances within and beyond 40–50 m separately. The regression coefficients *b* are −1.032 for the full data set (5–600 m), −1.563 for 5 to 50 m and −0.012 for 50 to 600 m.

The overall picture was a fast decreasing rate of colonizations from the centre and outwards, with a long tail of fewer establishments beyond 50 m (Fig. 2).

The mother colony had an estimated number of 10 000 capsules, based on a density of *c* 100 000 capsules per m^2^. Based on these values and the estimated number of spores per capsule (15 000±2500 [SE]), we calculated the total spore output to be within the range of 125–175 million spores.

The colonization rates (ratio of colonized pots) ranged from 50% to 100% at all 12 sampling stations within the central 10 m, with estimated densities between 141 and 439 spores/m^2^ among stations (Table 1, Table S1). There was no obvious difference in deposition pattern among directions (Fig. 2a). At 30 m, the colonization rates were intermediate and varied from 0 to 33% among the four stations, with an estimated density of 40±46 (SD) spores/m^2^. The colonization rates ranged from 0 to 5% among the 20 stations at 50 m to 600 m from the centre, with an estimated density of 2.3±2.6 to 5.8±4.0 spores/m^2^ among distances (averaged over stations in the four directions) (Table 1, Table S1). Inspection of the data in log-log format revealed that an inverse power function (*y* = *ax* ^−*b*^) with the slope *b* of −1.56 fitted quite well to the data up to 50 m (R^2^ = 0.80; p = 0.10), whereas the tail of the probability density function showed no relation to distance (Fig. 2b).

The estimation of the total number of spores deposited within a circle of 1 km according to the inverse power functions was calculated (equation 2) to 4.6 million, which is one to two orders of magnitude lower than the output (of 125–175 million).

At the first reference mire, situated 7 km from Jordbärsmuren, there were no colonizations. At the second reference mire, situated 36 km from Jordbärsmuren and 8 km from another translocated colony, 3 pots out of 233 were colonized, which corresponds to a colonization rate of 1% and an estimated spore density of 3 spores/m^2^.

## Discussion

The novel method of using suitable substrates as spore traps proved to be successful to measure realized dispersal (including establishment) at much longer distances than has been done before and should be considered further for these and other similar questions. Our result of surprisingly high colonization rates at distances up to 600 m show that species with diaspores larger than 20 µm can disperse and colonize efficiently (over short time scales) over considerable distances even if they are released close to the ground. Such a capacity facilitates a species to track transient patches in a dynamic landscape, where substrate patches may be separated by hundreds to thousands of meters [43]. Another interesting result was the rather even establishment from 50 meters and beyond. If this is a general pattern it has large implications for how we look upon connectivity and fragmentation for these kinds of organisms. It is also obvious that negative exponential models [14] underestimate dispersal distances.

### The representativeness of our model species

Our model organism inhabits open landscapes, on a substrate with no or sparse vascular plant vegetation, which facilitates dispersal compared to other species that releases their diaspores in dense vegetation or forests. In densely vegetated circumstances, a large proportion of the spores should become intercepted by vegetation, under reduced wind speeds that further hamper dispersal [44], [45] and may not reach above the canopy so a higher proportion will remain within the vicinity of the release point. Thus, our results may not be representative of all species. Another property that is important is the release height. It has been shown that trees which release their seeds from a height of tens of metres can disperse hundreds to thousands of metres [46]. However, in small bryophytes it might be more difficult for the diaspores to reach above the zone near the ground where the wind velocities are very low, cf. [27]. The spore diameter of our model species (20–30 µm) is in the upper part of the size distribution of moss spores (in a dataset of the mosses of Great Britain where most species have spores that are 10–20 µm [47], [48]), which indicates that most species may be even better dispersers than *Discelium*.

Direct measurements of long dispersal distances in bryophytes have not been conducted before. However, we were not surprised by the results given the fast colonization of recently exposed mineral soils several kilometres from known localities, which we have observed in the Umeå region (personal observations). Also recent results from studies on dispersal of mosses in the genus *Sphagnum* indicate very efficient dispersal [49]. Even if most bryophyte dispersal studies regarding diaspores of similar sizes have measured the dispersal only up to a few metres they have suggested that a high proportion of the spores is available for further dispersal, e.g. [21]. The two species *Atrichum undulatum* and *Bryum argenteum*, studied by Miles and Longton, are rather similar to *Discelium* since they have no specialized release mechanism, they have horizontal or pendulous capsules, peristomes and the spore diameters are 18 µm and 11 µm and only 2–5% and 7–13% of the spores were deposited within 2 m. However, in *Tortula truncata*, 70% of the spores were deposited within 2 m of a central colony of 46 sporophytes [22]. The upright spore capsule of *Tortula* lacks peristome, has a short seta and the spores are large (30 µm); traits that should account for the observed differences and hence make the spores of this species less likely to travel far.

Studies on the distribution patterns of epiphytes (in forests) suggest that they may be dependent on connectivity and are dispersal limited [14], [16]. Many of these epiphytes have diaspores that are larger than the spores of *Discelium*, wind speeds are much reduced within forest stands and the establishment may be a stronger bottleneck than for *Discelium*. This can also be the case for the small-spored forest fungi which might be strongly dispersal limited beyond 10–20 m as recently proposed by Norros et al. [19]. These differences can account for a higher dispersal limitation among the epiphytic species studied. Taken together, these studies support a substantial difference in dispersal and colonization capacity among bryophyte species.

### Dispersal distances and mechanisms

It has been suggested that dispersal of seeds can be biphasic with different mechanisms accounting for the short and long distance parts and that the probability density function in such a case could best be described by a mixed model [4]. This kind of pattern can be seen in our data, where the first part of the colonization pattern follows nicely a power-function with a slope of −1.56 while the second part shows no relation to distance (Fig. 2b). As a potential process generating the observed patterns we suggest that variation in wind speeds might bring spore clouds to different distances and that a portion of spores may be lifted up to higher air masses and become mixed there over larger areas before being deposited by precipitation or in still weather. One possible mechanism that could facilitate vertical transportation of diaspores is thermal upheaval catching the diaspores, even when the horizontal wind speed is low and transport them up into the atmosphere [34], but at higher wind speeds turbulence and other wind patterns become more important. The intermittency of winds might cause different dispersal patterns depending on the frequency and length of certain wind conditions, cf. [50]. Although a flat deposition curve may appear unrealistic (i.e. more spores reaching further and further distances) neither does a monotonic decline of the number of spores reaching each distance capture the details in dispersal process.

Another plausible explanation for the pattern found (of rather the even deposition density) is that there was a background deposition from other sources much further away. We find this less likely since the species is truly rare in the region where the experiments were performed and as colonization was only observed at one of the reference sites and there at a very low level. However, besides from understanding how spores disperse and distribute from point or patch sources, it is also important to understand to what extent background spore deposition rates in general are dense enough to support occasional establishment. This has implications for our ability to fully grasp the population dynamics and distribution patterns of this and other spore dispersed species [12], [17].

### The establishment rate and spore output

A comparison of the estimations of spore source strength and the total number of deposited spores (which resulted in a colonization) (125–175 million versus 4.6 million) shows that the number of spores predicted by the functions actually was available from the translocated colonies. Establishment was probably hampered by periodical droughts during the summer in the field. Based on our results from another study (unpublished) it is very likely that the colonization rates in this experiment would have been substantially higher (maybe twice as high) if we had brought the pots to optimal, moist conditions in a greenhouse. We also know that the substrate could be even better for *Discelium* if the pH of the clay would be somewhat lower (unpublished results). However, acknowledging these facts does not change the picture of that there was enough spores from the patch source to create the obtained result.

### Conclusion

As shown in our study, dispersal up to 600 m for diaspores larger than 20 µm is highly probable during one season, which should be vital for persistence of metapopulations in a landscape with ephemeral substrates. This study also suggests the possibility of a small patch source to build up a local airborne level of spores leading to an even deposition pattern over a large area. We hypothesize that the spores are divided into two fractions with rather different fates – either to fall close to the mother colony or to be uplifted to higher altitudes and possibly be dispersed far. For species being able to get their spores up in higher air masses, the background deposition rates will be more important for establishment at a newly disturbed place than the connectivity to other populations [12], [13], [23]. Instead the availability of suitable substrates and microclimatic conditions for establishment (time windows) may be the bottlenecks for such species [51]. The strength of the regional spore source will depend both on how common the species is at the landscape level, diaspore output and the ability to get the spores up in higher air masses. The results from this study should in principle be relevant for other organism groups with diaspores of similar or smaller size, but differences due to traits such as spore size, release conditions, habitat type and establishment rate among species are likely to be substantial. Not least the establishment probabilities might differ by many orders of magnitude among species and can account for different conclusions regarding realized dispersal capacity.

## Supporting Information

Table S1 The number of pots and colonization rates (ratio of colonized pots) for all 36 sampling stations.
(PDF)

## References

[1] Clobert J, Danchin E, Dhondt AA, Nichols JD, editors. Dispersal. Oxford: Oxford University Press 452.

[2] Bullock JM, Kenward RE, Hails RS, editors. Dispersal ecology. Malden: Blackwell Science 458.

[3] Cousens R, Dytham C, Law R (2008) Dispersal in plants: a population perspective. Oxford: Oxford University Press 221.

[4] Cain M, Milligan B, Strand A (2000) Long-distance seed dispersal in plant populations. Am J Bot 87: 1217–1227. 10991892

[5] Nathan R (2006) Long-distance dispersal of plants. Science 313: 786–788. 16902126 10.1126/science.1124975

[6] Fontaneto D, editor. Biogeography of microscopic organisms: is everything small everywhere? New York: Cambridge University Press. 365.

[7] Muñoz J, Felícisimo ÁM, Cabezas F, Burgaz AR, Martinez I (2004) Wind as a long-distance dispersal vehicle in the southern hemisphere. Science 304: 1144–1147. 15155945 10.1126/science.1095210

[8] Miller NG, McDaniel SF (2004) Bryophyte dispersal inferred from colonization of an introduced substratum on Whiteface Mountain, New York. Am J Bot 91: 1173–1182. 21653473 10.3732/ajb.91.8.1173

[9] Hutsemekers V, Dopagne C, Vanderpoorten A (2008) How far and how fast do bryophytes travel at the landscape scale? Divers Distrib 14: 483–492.

[10] Bremer P, Ott ECJ (1990) The establishment and distribution of bryophytes in the woods of the IJsselmeerpolders, the Netherlands. Lindbergia 16: 3–18.

[11] Cronberg N (2002) Colonization dynamics of the clonal moss *Hylocomium splendens* on islands in a Baltic land uplift area: reproduction, genet distribution and genetic variation. J Ecol 90: 925–935.

[12] Sundberg S, Hansson J, Rydin H (2006) Colonization of *Sphagnum* on land uplift islands in the Baltic Sea: time, area, distance and life history. J Biogeogr 33: 1479–1491.

[13] Hylander K (2009) No increase in colonization rate of boreal bryophytes close to propagule sources. Ecology 90: 160–169. 19294922 10.1890/08-0042.1

[14] Snäll T, Hagström A, Rudolphi J, Rydin H (2004) Distribution pattern of the epiphyte *Neckera pennata* on three spatial scales–importance of past landscape structure, connectivity and local conditions. Ecography 27: 757–766.

[15] Löbel S, Snäll T, Rydin H (2006) Metapopulation processes in epiphytes inferred from patterns of regional distribution and local abundance in fragmented forest landscapes. J Ecol 94: 856–868.

[16] Gjerde I, Blom HH, Lindblom L, Sætersdal M, Schei FH (2012) Community assembly in epiphytic lichens in early stages of colonization. Ecology 93: 749–759. 22690626 10.1890/11-1018.1

[17] Edman M, Kruys N, Jonsson B (2004) Local dispersal sources strongly affect colonization patterns of wood-decaying fungi on spruce logs. Ecol Appl 14: 893–901.

[18] Jönsson MT, Edman M, Jonsson BG (2008) Colonization and extinction patterns of wood-decaying fungi in a boreal old-growth *Picea abies* forest. J Ecol 96: 1065–1075.

[19] Norros V, Penttilä R, Suominen M, Ovaskainen O (2012) Dispersal may limit the occurrence of specialist wood decay fungi already at small spatial scales. Oikos 121: 961–974.

[20] Söderström L, Jonsson BG (1989) Spatial pattern and dispersal in the leafy hepatic *Ptilidium pulcherrimum* . J Bryol 15: 793–802.

[21] Miles CJ, Longton RE (1992) Deposition of moss spores in relation to distance from parent gametophytes. J Bryol 17: 355–368.

[22] Roads E, Longton R (2003) Reproductive biology and population studies in two annual shuttle mosses. The Journal of the Hattori Botanical Laboratory 93: 305–318.

[23] Sundberg S (2005) Larger capsules enhance short-range spore dispersal in Sphagnum, but what happens further away? Oikos 108: 115–124.

[24] Pohjamo M, Laaka-Lindberg S, Ovaskainen O, Korpelainen H (2006) Dispersal potential of spores and asexual propagules in the epixylic hepatic *Anastrophyllum hellerianum* . Evol Ecol 20: 415–430.

[25] Bullock JM, Shea K, Skarpaas O (2006) Measuring plant dispersal: an introduction to field methods and experimental design. Plant Ecol 186: 217–234.

[26] Aylor DE (1987) Deposition gradients of urediniospores of *Puccinia recondita* near a source. Phytopathology 77: 1442–1448.

[27] Gregory PH (1973) The microbiology of the atmosphere. 2nd edition. second ed. London: Leonard Hill. 377.

[28] Thomson FJ, Moles AT, Auld TD, Kingsford RT (2011) Seed dispersal distance is more strongly correlated with plant height than with seed mass. J Ecol 99: 1299–1307.

[29] Nathan R, Schurr F, Spiegel O, Steinitz O, Trakhtenbrot A, et al (2008) Mechanisms of long-distance seed dispersal. Trends Ecol Evol 23: 638–647. 18823680 10.1016/j.tree.2008.08.003

[30] Finlay BJ (2002) Global Dispersal of Free-Living Microbial Eukaryote Species. Science 296: 1061–1063. 12004115 10.1126/science.1070710

[31] During HJ (1979) Life strategies of Bryophytes: a preliminary review. Lindbergia 5: 2–18.

[32] Wilkinson DM, Koumoutsaris S, Mitchell EAD, Bey I (2012) Modelling the effect of size on the aerial dispersal of microorganisms. J Biogeogr 39: 89–97.

[33] Nathan R, Katul GG, Horn HS, Thomas SM, Oren R, et al (2002) Mechanisms of long-distance dispersal of seeds by wind. Nature 418: 409–413. 12140556 10.1038/nature00844

[34] Tackenberg O (2003) Modeling long-distance dispersal of plant diaspores by wind. Ecol Monogr 73: 173–189.

[35] Boros Ádám, Járai-Kolmlódi M, Zoltán T, Nilsson S (1993) An atlas of recent European bryophyte spores. Budapest: Scientia Publishing 321.

[36] Arnell HW (1875) De skandinaviska löfmossornas kalendarium. Akademiska afhandling. Uppsala: Uppsala universitet. 144.

[37] Shaw J, Allen BH (1985) Anatomy and Morphology of the Peristome in *Discelium nudum* (Musci: Disceliaceae). Bryologist 88: 263–267.

[38] Nyholm E (1989) Illustrated flora of Nordic mosses. Fasc. 2, Pottiaceae - Splachnaceae - Schistostegaceae. Copenhagen: The Nordic Bryological Society. 75–141.

[39] Smith AJE (2004) The moss flora of Britain and Ireland. Cambridge: Cambridge University Press. 1012.

[40] Hill MO, Bell N, Bruggeman-Nannenga MA, Brugués M, Cano MJ, et al (2006) An annotated checklist of the mosses of Europe and Macaronesia. J Bryol 28: 198–267.

[41] Bullock J, Clarke R (2000) Long distance seed dispersal by wind: measuring and modelling the tail of the curve. Oecologia 124: 506–521. 28308389 10.1007/PL00008876

[42] R Development Core Team (2011) A language and environment for statistical computing. R Foundation for Statistical Computing, Vienna, Austria. ISBN 3-900051-07-0, URL http://www.R-project.org/.

[43] Aune K, Jonsson BG, Moen J (2005) Isolation and edge effects among woodland key habitats in Sweden: Is forest policy promoting fragmentation? Biol Conserv 124: 89–95.

[44] Aylor DE, Ferrandino FJ (1985) Rebound of pollen and spores during deposition on cylinders by inertial impaction. Atmos Environ 19: 803–806.

[45] Pounden E, Greene DF, Quesada M, Contreras-Sanchez JM (2008) The effect of collisions with vegetation elements on the dispersal of winged and plumed seeds. J Ecol 96: 591–598.

[46] Greene D, Johnson E (1995) Long-distance wind dispersal of tree seeds. Can J Bot 73: 1036–1045.

[47] Hill MO, Preston CD, Bosanquet SDS, Roy DB (2007) BRYOATT: attributes of British and Irish mosses, liverworts and hornworts. Cambridge: Centre for Ecology and Hydrology 88.

[48] Lönnell N (2011) Wind dispersal of spores with focus on bryophytes. Plants & Ecology 2011/3: 1–37.

[49] Sundberg S (in press) Spore rain in relation to regional sources and beyond. Ecography doi: 10.1111/j.1600–0587.2012.07664.x.

[50] Aylor DE (1990) The role of intermittent wind in the dispersal of fungal pathogens. Annu Rev Phytopathol 28: 73–92.

[51] Halvorsen Økland R, Rydgren K, Økland T (2003) Plant species composition of boreal spruce swamp forests: Closed doors and windows of opportunity. Ecology 84: 1909–1919.

