# Supplementary material for: The Fate of the Missing Spores — Patterns of Realized Dispersal beyond the Closest Vicinity of a Sporulating Moss
Source: PLoS One. 2012 Jul 27;7(7):e41987. doi: 10.1371/journal.pone.0041987 (PMC3411459; doi:10.1371/journal.pone.0041987)
Supplement: Table S1 — The number of pots and colonization rates (ratio of colonized pots) for all 36 sampling stations. (PDF) [file pone.0041987.s001.pdf]

Supporting Information

Table S1 *The number of pots and colonization rates for all 36 sampling stations.*

Direction	Distance (m)	No of pots	Colonizable pots*	Colonizations†	Colonization rate	Density (spores/ m^2)
E	1	6	6	4	0.67	224
N	1	6	6	6	1	439
S	1	6	6	6	1	439
W	1	6	6	3	0.5	141
E	5	6	6	6	1	439
N	5	6	6	4	0.67	224
S	5	6	6	5	0.83	366
W	5	6	6	6	1	439
E	10	8	8	5	0.63	200
N	10	8	8	5	0.63	200
S	10	8	8	6	0.75	283
W	10	8	7	4	0.57	173
E	30	13	11	0	0	0
N	30	13	13	0	0	0
S	30	13	13	4	0.31	75
W	30	13	6	2	0.33	83
E	50	21	20	0	0	0
N	50	21	15	0	0	0
S	50	21	21	0	0	0
W	50	21	19	1	0.05	11
E	80	34	23	1	0.04	9
N	80	34	33	1	0.03	6
S	80	34	26	1	0.04	8
W	80	34	32	0	0	0
E	150	63	48	0	0	0
N	150	63	42	1	0.02	5
S	150	63	54	0	0	0
W	150	63	49	1	0.02	4
E	300	126	62	3	0.05	10
N	300	126	121	2	0.02	3
S	300	126	118	0	0	0
W	300	126	121	2	0.02	3
E	600	252	219	10	0.05	10
N	600	252	232	0	0	0
S	600	252	227	2	0.01	2
W	600	252	212	1	0	1

*Corresponding to (n) in equation 1,† Corresponding to (k) in equation 1.
